# Supplementary material for: MetaRibo-Seq measures translation in microbiomes
Source: Nat Commun. 2020 Jun 29;11:3268. doi: 10.1038/s41467-020-17081-z (PMC7324362; doi:10.1038/s41467-020-17081-z)
Supplement: Supplementary file 10 — Supplementary Data 7 [file 41467_2020_17081_MOESM10_ESM.zip › File2/Confidence_High_Taxonomy/396514_out.krona.html]

Javascript must be enabled to view this page.

members
magnitude
magnitudeUnassigned
count
unassigned
taxon
rank

396514\_out

12

2
superkingdom
12

12
phylum
1239

12
class
186801

12

SRS015431\_contig\_number\_71919SRS018427\_contig\_number\_24436SRS020869\_contig\_number\_18465SRS1041033\_contig\_number\_4883
4
186802
order

541000
family
1

1263
genus
1


SRS148159\_contig\_number\_47040
1816688
species
1

family
186803
7

33042
genus
1

species

SRS052697\_contig\_number\_16410
116085
1

6
572511
genus

6
species
765821

SRS015217\_contig\_number\_30296SRS015578\_contig\_number\_2217SRS019068\_contig\_number\_66974SRS1054691\_contig\_number\_18951SRS146813\_contig\_number\_17838SRS148721\_contig\_number\_51261
